# Supplementary material for: Helping Women Suffering from Drug Addiction: Needs, Barriers, and Challenges
Source: Int J Environ Res Public Health. 2022 Oct 28;19(21):14039. doi: 10.3390/ijerph192114039 (PMC9656705; doi:10.3390/ijerph192114039)
Supplement: Supplementary file 1 [file ijerph-19-14039-s001.zip › ijerph-1940620-supplementary.pdf]

Table S1: Data on barriers, needs, and challenges to addiction treatment based on research among women who use psychoactive substances

Study (author, year)	Brief description	Sample size (n)	Barriers	Needs	Challenges
Bungay, 2013	Study among British Columbia women who use drugs	n=63	Extreme poverty.<br>The need to conceal drug use problems in order to receive full or better health care in medical facilities.<br>The shame associated with being a person in crisis.	More facilities exclusively for women with addiction problems (without the presence of male patients), as well as medical facilities for pregnant women or women with children.<br>Under-services for women over 40.	Stigmatization and discrimination.<br>Hierarchicality, i.e., exploitation of position: staff-women with addiction.<br>Ageism.
Edelman et al., 2013	Barriers to accessing health services among English women drug users	n=20	Low sense of self-worth and self-efficacy; low acceptance of addiction as a disease; suppression of depression; high levels of anxiety.	Therapy for coping with stress, anxiety, low self-esteem and self-efficacy, suppression of depression.<br>Modification of the therapy program.<br>Sensitivity of staff when working with women with drug use problems.	Impoverished social relationships increasing respondents' reluctance to meet with specialists.<br>Self-protective measures against loss of "good" well-being. Social stigma.
Mehrjerdi et al., 2013	A study among Iraqi women using amphetamines and heroin at the same time.	n=82	Loneliness.<br>Low socioeconomic statute.<br>Lack of knowledge about the dangers of using psychoactive drugs.	Addiction prevention.<br>Harm reduction and addiction treatment programs for women.<br>Research on the determinants of drug use among women.	Early drug initiation.<br>High risk of health complications-especially mental health-associated with concurrent use of sedative and stimulant drugs.

					The risk of multiple risky sexual behaviors associated with the need to obtain drugs.
Labmdin et al., 2013	Inequalities in harm reduction services among Tanzanian male and female drug users.	n=1098	High levels of sex-related risk behavior, anxiety, depression and sexual abuse.	Scientific initiatives on the problems of women who use drugs.<br><br>Strengthening harm reduction services for women.	Low access to preventive and therapeutic services.<br><br>Violence.<br><br>High risk of HIV infection.<br><br>Fear of social consequences.
Otiashvili, et al., 2013	Research on access to treatment for women drug addicts in the Republic of Georgia.	n=55	Extreme stigmatization<br><br>Social hostility.<br><br>Intolerance from the family.<br><br>Lack of gender-related services, judgment by medical personnel.<br><br>Repression of drug users.<br><br>Paid treatment programs.<br><br>Guilt and low self-esteem.	Public education about addiction as a medical condition.<br><br>Educate health services about the needs of women substance users.<br><br>Introducing services aimed at women.	Changes in drug policy.<br><br>Socio-cultural determinants of negative attitudes toward drug users - especially toward women.
Bairan et al., 2014	A study of the needs of suburban US women who abuse drugs.	n=65	Financial problems, lack of transportation, difficulties in accessing social benefits and health care.<br><br>Excessive bureaucracy.	Holistic determination of the health needs of women with addiction problems.<br><br>Implementation of appropriate social, health and care interventions.	The so-called domino effect: difficulties in returning to fulfilling social roles related to previous experiences.

			Marginalization, stigmatization.	Appropriate preparation of care and medical services.	
Edelman et al., 2014	A study of sexual health risks among English women with substance abuse.	n=77	Difficulty remembering reporting deadlines.<br><br>Reluctance to disclose their problems.<br><br>A low sense of self-worth.<br><br>Use of psychoactive substances to suppress depressive states and high levels of anxiety.	Educating medical personnel on how to know how to work with a patient with a possible history of sexual assault, and at risk of pregnancy and past or present sexually transmitted diseases.<br><br>Activating women substance users to stay in touch with medical and social services.	Access to doctors' offices and counseling related to both treatment of substance abuse problems and sexual health problems.<br><br>Access to related institutions, e.g., related to anti-violence, sexual assault.
Powelson et al.,<br>2014	A study of the health needs of U.S. women amphetamine users.	n=298	Lack of awareness of one's own health needs, with a subjective lack of need for medical services.<br><br>Stigmatization.	Unmet health care needs in three areas: chronic health problems, dermatological problems and preventive women's health care.<br><br>Harm reduction interventions.	Education about health needs, both in terms of medical assistance to this group and in terms of introducing appropriate preventive interventions.<br><br>Introducing empathetic, non-stigmatizing health care providers who are willing to fully commit to helping this group.
Davis, et al., 2015	Research on the determinants of the	n=102	Discrimination, marginalization and stigma	Therapists' high cultural competence, empathy,	High professional preparation of therapists.

	therapeutic alliance necessary for therapy, conducted among African-American women substance abusers in the US .		associated with being a female substance abuser.<br><br>Internalized feelings of powerlessness and shame.	unconditional positive regard and authenticity (Rogers' triad).	Egalitarianism.
Khuat, et al., 2015	Social context, diversity, and risk among Vietnamese women intravenous drug users.	n=403	Stigma.<br><br>Low access to HIV testing, lack of knowledge about sexually transmitted diseases and viral hepatitis.	Education on HIV transmission.<br><br>Differentiated strategies in accessing women in need of help with drug use.	Intensification of prevention programs to prevent blood-borne infections.<br><br>Psychological support, counseling, education in family planning and parenting skills.
Haritavorn, 2016	A study on the role of motherhood in shaping lives among female intravenous drug users in Thailand.	n=30	Economic constraints.	Education about procreation and options to protect against unplanned pregnancy.<br><br>Low coping skills to deal with numerous emotional challenges, from anxiety to guilt, shame.	Maintaining or improving the identity of a good mother.
Lee, Boeri, 2017	Survey among U.S. women with substance abuse problems on:<br><br>1. the stigma of drug use;	n=20	Stigmatization.<br><br>Low competence in dealing with unpleasant emotions.	Social skills training.<br><br>Education on alternatives to drugs for dealing with unpleasant emotions.	Establish appropriate centers for women with adequate therapy and well-trained therapeutic staff.

	2. perceptions of normalcy among women drug users;<br><br>3. barriers and challenges to improving their situation.		Subjective sense of "benefit" from substance use.<br><br>Low effectiveness of drug treatment and treatment programs not tailored to women's needs.	Greater social integration with people outside the substance abuse community.	
Sharma, et al., 2017	A study of sexual and reproductive health among women drug users in India.	n=48	Shame in discussing intimate problems.<br><br>Stigmatization of women who abuse drugs.<br><br>The high cost of treatment.	Health education.<br><br>Addiction prevention.<br><br>Establish interventions targeting women that act as a "one-stop shop" for a comprehensive package of health services: substitution therapy, detoxification and rehabilitation programs, etc.	Low level of need for health care services.<br><br>Very low education, with a lack of knowledge about the consequences of drug use and sexual health.
Ayon, et al., 2018	A study of the experiences of Kenyan women who inject drugs with regard to access to HIV, harm reduction, and sexual and reproductive health services.	n=45	Stigmatization.<br><br>Long distances to providers.<br><br>Lack of confidentiality.<br><br>Fees for using support services.<br><br>Lack of knowledge of medical facilities among drug users.	Educating women about their health and rights.<br><br>Connecting women with health care facilities.<br><br>Introducing harm reduction interventions and sexual and reproductive health services.	Sensitizing providers to the needs of women who inject drugs.<br><br>Building an atmosphere of trust.

			Lack of communication with health care providers.<br>Lack of understanding of women's needs by providers.		
Lambdin, et al., 2018	Survey of unmet health care needs among incarcerated U.S. women drug users.	n=624	Marginalization.<br>Homelessness.<br>Feeling unsafe in one's living environment.<br>Stress.	Unmet health care needs.<br>Gap in support programs for female drug users.	Avoiding seeking help in public institutions (hospitals, schools) because of fears of mistreatment for having been incarcerated in the past.<br>Poor mental health.
Mburu, et al., 2018	A study of the stigmas faced by women who inject drugs in Kenya.	n=45	Self-stigmatization.<br>Gender stigma - being a female injecting drug user.<br>Stigma from the family.<br>Stigmatization of HIV-infected people by other drug users.	Educating women about their health and rights.<br>Introducing harm reduction interventions.<br>Therapeutic work on improving self-esteem.<br>Introducing training for providers on the problems and needs of women who use drugs.	Isolation.<br>Exclusion.<br>Discrimination and stigma in health care facilities.
Staton, et al., 2018	A study of the correlates of injection drug use among	n=400	Mental health problems.	Improving the dire economic situation.	Prescription of painkillers by rural doctors.

	women living in rural Appalachia.		Poor access to support institutions.<br><br>Experiencing victimization.	Harm reduction activities.<br><br>Prevention of addiction and HCV infection.	Marketing efforts to distribute painkillers.<br><br>Limited treatment options.
Ayon, et al., 2019	A study of the needs of Kenyan female injecting drug users and harm reduction stakeholders.	n=45	Low economic status.<br><br>Low accessibility to sexual health services.<br><br>Lack of access to health services for women with addiction problems serving prison sentences.<br><br>Stigmatization of women who use drugs.	Education on family planning.<br><br>Lack of funds to hire qualified nurses.<br><br>Harm reduction, including syringe and needle exchange programs.<br><br>Training for staff to become competent to provide services to women who inject drugs.	Integrating drug treatment, family planning education and sexual health services with other community services.<br><br>Changing social norms in the perception of women.
Boroumandfar, et al., 2020	A study of factors influencing Iranian women's decision to stop using drugs.	n=30	Stigmatization.<br><br>Lack of awareness of existing social support services.<br><br>Lack of special rehabilitation centers for women.<br><br>Rejection by the family and conflict between family members.	Lack of offers to support the sustainability of drug therapy after treatment is completed.<br><br>Education on life skills.<br><br>Family sessions to help women understand and be accepted after discharge from rehab centers.	Creating special rehabilitation centers for women to help them meet their specific needs.<br><br>Creating free drug treatment.<br><br>Reducing family disputes.

			Addiction of other family members.<br><br>High costs of drug treatment.		
Collins, et al., 2020	Investigating the impact of housing crises on the overdose risk of Canadian women substance abusers.	n=35	Socio-economic marginalization.<br><br>Stigmatization.<br><br>Racial and gender-based violence.<br><br>Shame.	Taking care of women's needs.<br><br>Conducting safer community interventions in low-income housing.<br><br>Improving living conditions.	Gender inequality: culturally perpetuated image of women as docile, subservient, subordinate to men.<br><br>Subjective treatment of women who abuse substances.
Shirley-Beavan, et al., 2020	Barriers to using harm reduction services among Spanish women drug abusers.	n=12	Social stigma.<br><br>Limited access to diagnosis, treatment and successful health outcomes.<br><br>Gender-based violence.<br><br>Lack of services that are tailored to deal with violence for women drug users.<br><br>Lack of services focused on the needs of women, particularly sexual and reproductive health and care services.	Training for health professionals on human rights and medical ethics.<br><br>Establishing rehab facilities that meet the needs of women.<br><br>Conduct that respects human rights.	Structural barriers to accessing harm reduction services.<br><br>Limited resources that can improve the situation of women in drug abuse crisis.<br><br>Stigma and discrimination by health professionals, denial of care, provision of substandard care, physical and verbal abuse, longer waiting periods, transfer of care to younger colleagues,

					and disclosure and lack of confidentiality.
Akré, et al., 2021	Exploring the relationship between social stability and access to health care services among U.S. female drug users.	n=538	Unfavorable socioeconomic conditions.<br><br>Paid therapy.	High level of unmet health needs.<br><br>Treatment programs to meet the specific needs of women drug users.	Improving social factors such as housing stability and personal security for women.<br><br>Creating safe spaces by health systems to allow women with limited social stability to access care free of charge.
Yona, et al., 2021	A study of the experiences of Indonesian women living with HIV during recovery from drug treatment.	n=22	Stigmatization.<br><br>Limited number of rehabilitation centers.<br><br>Lack of therapeutic offerings and programs specifically tailored to women's unique needs.	Family support, especially from the husband.<br><br>Lack of knowledge about addiction and HIV transmission.<br><br>Counseling and services run by women for women.	Treatment policies and services that are sensitive to women's experiences and consider their needs.

Source: Own compilation
